# The impact of different intensities and domains of physical activity on analgesic use and activity limitation in people with low back pain: A prospective cohort study with a one‐year followup

**DOI:** 10.1002/ejp.1987

**Published:** 2022-06-16

**Authors:** Thomas G. Patterson, Paula R. Beckenkamp, Manuela Ferreira, Adrian Bauman, Ana Paula Carvalho‐e‐Silva, Lucas Calais Ferreira, Paulo H. Ferreira

**Affiliations:** ^1^ Faculty of Medicine and Health The University of Sydney, Discipline of Physiotherapy, Sydney School of Health Sciences Camperdown New South Wales Australia; ^2^ Faculty of Medicine and Health The University of Sydney, Sydney Musculoskeletal Health, School of Health Sciences, The Kolling Institute of Medical Research St Leonards New South Wales Australia; ^3^ Faculty of Medicine and Health The University of Sydney, Public Health, Sydney School of Public Health Camperdown New South Wales Australia; ^4^ Twins Research Australia Unit The University of Melbourne, School of Population and Global Health Carlton Victoria Australia

## Abstract

**Background:**

Analgesics are the most common form of managing low back pain (LBP). No previous study has examined which domains and intensities of physical activity are most beneficial in reducing the frequency of analgesic use for LBP and its related activity limitation.

**Methods:**

This cohort study forms part of the AUstralian Twin low BACK pain study, investigating the impact of physical activity on LBP. Information on demographics, LBP and health‐related factors, including physical activity, were collected at baseline. Data on the total counts of analgesic use and activity limitation for LBP were collected weekly for one‐year. Negative binomial regression models were conducted separately for each type of physical activity. Results were presented as incidence rate ratios (IRRs) and 95% confidence intervals (CIs).

**Results:**

From an initial sample of 366 participants, 86 participants reported counts of analgesic use and 140 recorded counts of activity limitation across the follow‐up period. The negative binomial regression models for analgesic use counts indicated that engagement in moderate‐vigorous physical activity was protective for use of analgesics (IRR 0.97, 95% CI 0.96–0.99), while physical workload was associated with greater use (IRR 1.02, 95% CI 1.01–1.05). No other significant relationships were observed for the other measures of physical activity. For activity limitation counts, engagement in leisure activity was associated with less counts of activity limitation (IRR 0.94, 95% CI 0.81–0.99), while greater amounts of sedentary time was associated with higher counts (IRR 1.04, 95% CI 1.01–1.09). No other significant relationships were observed for the other measures of physical activity.

**Conclusions:**

Our findings highlight the potential importance of supporting engagement in moderate–vigorous and leisure physical activity as well as minimizing sedentary time and physical workload to reduce the risk of activity limitation and the need for analgesic use in people with LBP.

**Significance:**

We examined which domains and intensities of physical activity are most beneficial in reducing the frequency of analgesic use for low back pain and its related activity limitation. Engaging in moderate–vigorous and leisure physical activity as well as minimizing sedentary time and physical workload has the potential to reduce the risk of activity limitation and the need for analgesic use in people with low back pain.

## 1 INTRODUCTION

Low back pain (LBP) is a common condition, with a lifetime prevalence as high as 84% (Hoy et al., 2012), affecting more than 560 million people worldwide (Hartvigsen et al., 2018; James et al., 2018; Vos et al., 2020). LBP is also responsible for over 60 million years lived with a disability annually (Buchbinder et al., 2018; James et al., 2018; Maher et al., 2017; Walker, 2000), placing it as the leading cause of disability globally (Collaborators GDaIIaP., 2016; James et al., 2018). In Australia, the direct costs associated with the management of LBP are approximately $5 billion per annum (Martin et al., 2013), with prescription and over‐the‐counter analgesics accounting for a significant proportion of this cost (Becker et al., 2010; Gore et al., 2012; Martin et al., 2013; Walker et al., 2003). Furthermore, when additional costs such as loss of wages, disability subsidy and decreased productivity are considered, the economic burden almost doubles (Walker et al., 2003). The individual and economic burden of LBP can be attributed to the recurrent nature of the condition (da Silva et al., 2019; Foster, 2011; Hartvigsen et al., 2018; Walker et al., 2003), with over 60% of people experiencing at least one reoccurrence of LBP within 12 months following an episode (da Silva et al., 2019) and more than 50% also reporting limitations when performing daily activities or having to seek care due to their LBP (da Silva et al., 2019).

Analgesics such as paracetamol, Non‐Steroidal Anti‐Inflammatory Drugs (NSAIDS) and opioids are the most common pain medications prescribed by health professionals to manage LBP (Buchbinder et al., 2021; Goertz et al., 2021; Rodondi et al., 2018; Wilk et al., 2010). The potential for misuse and growing safety concerns associated with these analgesics is well documented (Cairns et al., 2019; Larson et al., 2005; Machado et al., 2015; Migliorini et al., 2021; Roberts et al., 2016; Schnitzer et al., 2004; Shaheed et al., 2021; Sistanizad & Peterson, 2013; Tucker et al., 2020) and recent evidence has now emerged that paracetamol, NSAIDS and opioids provide no or minimal clinical benefit to people with LBP (Machado et al., 2015; Migliorini et al., 2021; Schnitzer et al., 2004; Shaheed et al., 2021; Tucker et al., 2020; van der Gaag et al., 2020). As a result, many clinical guidelines worldwide prefer that LBP is managed without analgesics and encourage individuals to keep active and avoid prolonged periods of sedentary time (Buchbinder et al., 2018; Mishriky et al., 2020; Oliveira et al., 2018).

Different types and intensities of physical activity have been previously shown to have protective or harmful effects on LBP (Heneweer et al., 2009; Heneweer et al., 2012; Heuch et al., 2017; Kim et al., 2014; Kwon et al., 2011a; Shiri & Falah‐Hassani, 2017; Solovev et al., 2020; Zadro et al., 2017). Meeting the World Health Organization physical activity guidelines (Zadro et al., 2017) and engaging in moderate amounts of leisure physical activity time have been shown to be protective against chronic LBP (Shiri & Falah‐Hassani, 2017; Solovev et al., 2020), whereas longer durations of total physical activity minutes per week (Heneweer et al., 2009; Heneweer et al., 2012; Solovev et al., 2020), increased sedentary time (Heneweer et al., 2009) and strenuous work‐related physical activity have shown to increase the risk of chronic LBP (Heuch et al., 2017). In addition, community‐dwelling older adults who engage in low amounts of moderate–vigorous physical activity per day have greater numbers of drug prescriptions annually, compared to those engaging in high amounts of moderate–vigorous physical activity (IRR = 1.53 [1.18–2.00]) (Simmonds et al., 2014). However, there is still uncertainty about which specific intensities and domains of physical activity are most beneficial in reducing the frequency of analgesic use for LBP and its related activity limitation.

The aim of this study was to assess the relationship between different domains (e.g. leisure, transport, household, work related), and intensities (e.g. moderate, vigorous) of physical activity assessed via the device and self‐reported questionnaires, and the frequency of analgesic use and activity limitation in people with LBP.

## 2 METHODS

### 2.1 Study sample and data collection

This cohort study forms part of the Australian Twin low BACK pain (AUTBACK) study (Pinheiro et al., 2016), a longitudinal observational study examining hereditary and lifestyle factors associated with LBP. In the AUTBACK study, participants were recruited from Twins Research Australia (TRA), a large non‐profit organization that maintains a nation‐wide database of over 45,000 twin pairs of all zygosity types and ages (Murphy et al., 2019). Recruitment for the AUTBACK cohort occurred from October 2015 to June 2019. Information on demographics, anthropometrics, LBP status (severity, disability related and length of symptoms) and health‐related factors (physical activity, depression, anxiety, stress and sleep quality) were collected at baseline through online self‐reported questionnaires. Additional data on physical activity were obtained with accelerometers. Further details regarding the recruitment and data collection procedures used in the AUTBACK study can be found elsewhere (Pinheiro et al., 2016). All recruitment and data collection procedures used in the AUTBACK study were approved by both the University of Sydney Human Research Ethics Committee and the TRA under Project Number 2015/407, and participants provided informed written consent.

### 2.2 Inclusion and exclusion criteria

We included adults from the AUTBACK study, irrespective of whether they did or did not have a history of LBP (recorded at baseline) and considered both individuals who did and did not report analgesic use or activity limitation for their LBP over 1 year in this study. Eligible participants required internet access via computer or smartphone and an active email account. Individuals with any self‐reported serious spinal pathology (e.g. inflammatory, metastatic or infectious disease of the spine), pregnant women, and those who had undergone spinal surgery in the last 12 months were not eligible to take part in the AUTBACK study.

### 2.3 Design

This study employed a prospective cohort design. We aimed to identify prognostic markers between different domains and intensities of physical activity and the total frequency (counts) of analgesic use and activity limitation in people with LBP during a 1‐year period.

### 2.4 Outcome variables

The primary outcome was the total frequency (counts) of self‐reported analgesic use for LBP assessed on a weekly basis during a 1‐year period. The secondary outcome was the total frequency (counts) of self‐reported activity limitation associated with LBP assessed on a weekly basis during a 1‐year period. One count of analgesic use for LBP was defined as the participant responding ‘Yes’ to the question: ‘Did you take medications (non‐opioids, weak opioids, strong opioids, anti‐depressants, natural pain relievers or others) for your LBP in the last week?’ One count of activity limitation was defined as the participant indicating Yes to the question: “Was the LBP bad enough to limit your activity (work, social, sports, hobbies, intimacy or chores) in the last week?” If the participant responded ‘No’ to analgesic use or activity limitation in the last week, this was recorded as a count of zero. Both the primary and the secondary outcomes were recorded weekly over 1 year via specifically designed automated SMS messages sent at the preferred time indicated by the participant. The total frequency (counts) was summed for each week and totalled for 1‐year post‐baseline.

### 2.5 Physical activity

Objective data on physical activity were obtained with an accelerometer (Actigraph GT1M/GT3X, ActiTrainer, ActiGraph, LLC, Pensacola, FL, USA), which was posted to participants on a pre‐paid return envelope along with the instructions for wearing. The Actigraph recorded data on body movement, activity counts, energy expenditure and body position across 7 days at baseline, which was validated by the research team upon return of the Actigraph. Only those with a complete 7 days worth of data, with a minimum of 8 h of wear time on each day were included in the analysis. The Actigraph generated data on the duration of moderate–vigorous physical activity and sedentary time. The use of accelerometers, such as the Actigraph GT1M/GT3X, has shown to be one of the most valid, reliable, accurate and sensitive instruments for assessing physical activity (Colbert et al., 2011; Ellis et al., 2014; Warren et al., 2010).

Self‐reported data on physical activity domains including household, transport, leisure and work measured as MET minutes per week (MET min/week) were obtained through the long version of the International Physical Activity Questionnaire (IPAQ‐long) at baseline. The IPAQ‐long assesses the frequency and duration of physical activity in the aforementioned domains and has acceptable measurement properties (Hagströmer et al., 2006). Additionally, the physical workload was recorded using the Physical Workload Index questionnaire (Hollmann et al., 1999), which has shown to be a valid and reliable tool to assess the frequency of people engaging in different postures and tasks at work.

### 2.6 Covariates

Covariates were chosen based on the potential association between physical activity and analgesic use and activity limitation for LBP. Previous research has shown that activity limitation and analgesic use for LBP can be associated with factors such as pain intensity (Monticone et al., 2021; Oliveira et al., 2019; Patterson et al., 2021; Severeijns et al., 2001; Sturgeon, 2014) and duration (Hayden et al., 2010; Sribastav et al., 2018), depression (Grabovac & Dorner, 2019; Monticone et al., 2021; Oliveira et al., 2019; Pinheiro et al., 2015; Severeijns et al., 2001; Sturgeon, 2014), anxiety (Grabovac & Dorner, 2019; Monticone et al., 2021; Oliveira et al., 2019; Severeijns et al., 2001; Sturgeon, 2014), stress (Grabovac & Dorner, 2019; Severeijns et al., 2001; Sturgeon, 2014), sleep quality (Ho et al., 2021; Kovacs et al., 2018; Patterson et al., 2021), level of disability (Ferreira et al., 2010; Monticone et al., 2021; Oliveira et al., 2019; Severeijns et al., 2001; Sturgeon, 2014), age (Mannion et al., 2013), gender (Ferreira et al., 2010; Ho et al., 2021; Mannion et al., 2013) and body mass index (BMI) (Hashimoto et al., 2018; Miura et al., 2019; Sribastav et al., 2018; Stevans et al., 2021). Therefore, the confounding effects of these aforementioned factors on analgesic use and activity limitation were accounted for in the study analysis.

### 2.7 Sleep quality

Participants' sleep quality was assessed using the Pittsburgh Sleep Quality Index (PSQI). The PSQI is a valid and reliable 18‐item self‐report questionnaire which assesses sleep quality in seven domains as follows: subjective sleep quality, sleep latency, sleep duration, habitual sleep, sleep disturbances, use of sleeping medication and daytime dysfunction (Buysse et al., 1989; Buysse et al., 2008). The total score is composed of the sum of scores for these seven domains and ranges from 0 to 21, with scores higher than 5 points regarded as poor sleep quality (Buysse et al., 1989).

### 2.8 Depression, anxiety and stress

The short form of the Depression Anxiety Stress Scale (DASS‐21) was used to assess symptoms of depression, anxiety and stress. The DASS‐21 is composed of 21 items and is a valid quantitative measure of symptoms of depression, anxiety and stress (Henry & Crawford, 2005). Normal scores range from 0 to 9 for depression, 0 to 7 for anxiety and 0 to 14 for stress (Henry & Crawford, 2005), with higher scores indicating increased severity of depression, anxiety or stress (Henry & Crawford, 2005).

### 2.9 Disability

The Roland Morris Disability Questionnaire (RMDQ) was used to assess physical disability related to LBP (Roland & Morris, 1983). The RMDQ is a valid measure of disability and consists of 24 items representing physical activities of daily living that are likely to be affected by LBP (Macedo et al., 2011; Roland & Fairbank, 2000). Scores range from 0 to 24 with higher scores representing higher levels of disability due to LBP (Roland & Fairbank, 2000).

### 2.10 Pain intensity

The Numeric Pain Rating Scale (NPRS) is a valid, reliable, one‐dimensional numeric measure of pain intensity in adults and was used to collect the average pain intensity in the last week (Hawker et al., 2011). Responses ranged from 0 to 10, with higher scores indicating greater pain intensity (Ferreira‐Valente et al., 2011).

### 2.11 Data analysis

Descriptive statistics were conducted for all variables. Our primary outcomes were analysed using negative binomial regression models, which take into account positively skewed and over‐dispersed data from recurrent events (Allison & Waterman, 2002; Hilbe, 2011; Lawless, 1987; Ver Hoef & Boveng, 2007). As a result of the non‐linearity of the negative binomial distribution, the regression coefficients (B) are not directly interpretable; therefore, the incident rate ratios (IRRs) were presented (Allison & Waterman, 2002; Hilbe, 2011; Lawless, 1987; Ver Hoef & Boveng, 2007).

Multiple negative binomial regression models were fitted to assess the association between physical activity and episodes of analgesic use and activity limitation.

Models were fitted separately for each different domain and intensity of physical activity and were analysed for self‐reported and device‐based measures, both continuously and categorically. Based on data distribution, STATA statistical software generated three tertiles for the categorical variables, with high and middle categories compared to the low reference group.

The variables of sleep quality, depression, anxiety, stress, disability and pain intensity were dichotomized for the purposes of the analysis and achievement of appropriate convergence in negative binomial regression models. The cut‐offs for dichotomization were based on previous studies for each variable: sleep quality ≥6 (Buysse et al., 1989), depression ≤9 (Henry & Crawford, 2005), anxiety ≤7 (Henry & Crawford, 2005), stress ≤14 (Henry & Crawford, 2005), disability ≤5 (Kuijer et al., 2005) and pain intensity ≥3 (Hallegraeff et al., 2021). Each model was adjusted for covariates (age, BMI, pain intensity, stress, sleep quality and history of pain) that were found to be significantly associated (p < 0.1) with both predictors and outcomes in univariate models. Additionally, models were adjusted for the potential influence of using twin pairs in this cohort study through use of the VCE command in STATA. The remaining covariates (anxiety, depression, disability and gender) were not included in the adjusted models as they recorded *p* values >0.1. The level of significance was set at 0.05 for estimates of association in the negative binomial models and results were presented as IRR and 95% confidence intervals (CI). Data analyses were performed using STATA statistical software Version 15 (StataCorp. 2017. Stata Statistical Software: Release 15. College Station, TX: StataCorp LLC).

## 3 RESULTS

### 3.1 Sample characteristics

**FIGURE 1:**
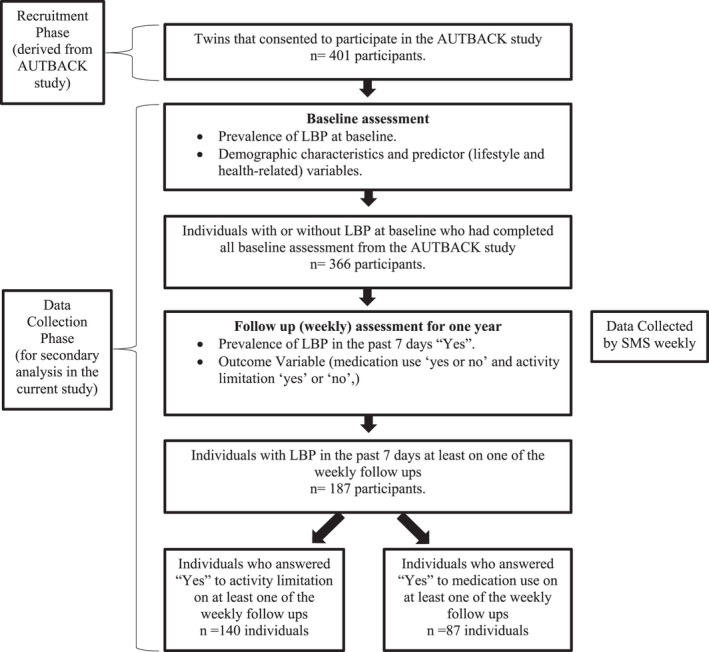
Flow of participants through the study

A total of 334 individuals fulfilled the criteria for this study and were included in the analysis. Out of these, 160 participants reported LBP at baseline and 174 were symptom free (Table 1). The majority was female (73%) and the mean age (± standard deviation) across the sample was 56.5 (±5.6 years). A total of 86 participants recorded at least one count of analgesic use to manage their LBP over the follow‐up period (Table 1). Additionally, 140 participants recorded at least one count of activity limitation across the 1‐year follow‐up due to their LBP (Table 1). The average number of counts of analgesic use and activity limitation during the 1‐year follow‐up was 8.8 (±5.9) and 6.7 (±4.5) weeks respectively (Table 1). The average number of counts for both analgesic use and activity limitation were higher in those experiencing LBP at baseline (10.1 ± 5.4 and 7.5 ± 5.2 respectively) compared to those without (8.2 ± 6.9 and 6.5 ± 4.4 respectively) (Table 1). In regard to physical activity, individuals who were not experiencing LBP at baseline, were on average less sedentary, had lower levels of physical workload, engaged more in leisure and transport activity and spent less time in household and work activity compared to individuals who were experiencing LBP at baseline (Table 1). The flow of participants through the study is shown in Figure 1.

**TABLE 1:** Study characteristics

	Total sample		LBP at baseline		No LBP at baseline	
	*n*	Mean (SD) or %	*n*	Mean (SD) or %	*n*	Mean (SD) or %
Demographics						
Age	334	56.5 (5.6)	160	56.1 (5.1)	174	56.8 (6.2)
Males	90	27%	42	26%	49	28%
Females	244	73%	118	74%	125	72%
Outcome variables						
Medication use counts	87	8.8 (5.9)	54	10.1 (5.4)	43	8.2 (6.9)
Activity limitation counts	140	6.7 (4.5)	78	7.5 (5.2)	62	6.5 (4.4)
Physical activity measures						
Moderate–vigorous physical activity (min/week)	329	175 (13)	158	158 (10)	171	188 (17)
Sedentary behaviour (min/week)	327	3355 (628)	155	3411 (492)	172	3297 (773)
Physical Workload (scores ranging 0–62)	281	9.8 (1.28)	150	10.7 (1.05)	131	9.2 (1.88)
Leisure domain physical activity (MET min/week)	334	729 (31)	160	704 (27)	174	777 (41)
Transport domain physical activity (MET min/week)	334	330 (26)	160	310 (24)	174	341 (37)
Household domain physical activity (MET min/week)	334	967 (20)	160	1011 (18)	174	751 (29)
Work domain physical activity (MET min/week)	334	240 (45)	160	594 (43)	174	5 (51)

Abbreviations: n, number of participants; SD, Standard deviation.

### 3.2 Physical activity and analgesic intake associated with LBP

Results from the negative binomial models showed that an increased time spent in moderate–vigorous physical activity was significantly associated with a lower number of analgesic use counts for LBP (IRR 0.97, 95% CI 0.96–0.99) (Table 2). These results were confirmed when participants in the middle (IRR 0.80, 95% CI 0.61–0.95) and in the high tertile of moderate–vigorous physical activity (IRR 0.74, 95% CI 0.60–0.89) were compared to the low tertile (Table 2). The physical workload was also found to be significantly associated with the number of analgesic use counts for LBP, with increased engagement in higher physical workload tasks being associated with a higher number of analgesic use counts (IRR 1.02, 95% CI 1.01–1.05) (Table 2). These results were further established when participants in the middle (IRR 1.08, 95% CI 1.01–1.13) and in the high (IRR 1.11, 95% CI 1.03–1.24) tertiles were compared to those in the low tertile (Table 2). In regard to sedentary time, results demonstrated that the association between sedentary time and the number of analgesic use counts was not significant when measured continuously or categorized across the tertiles (Table 2). Additionally, across the variety of physical activity domains (leisure, transport, household and work), no associations were found to be significant when measured continuously and across the tertiles (Table 2).

**TABLE 2:** The relationship between intensity and domain of physical activity and analgesic use episodes for LBP (*n* = 87)

Explanatory variable	Volume	IRR	95% CI	*p*
Sedentary behaviour	Continuous	1.06	0.93–1.11	0.07
	Low	*reference*		
	Middle	1.13	0.98–1.49	0.08
	High	1.18	0.55–3.01	0.08
By intensity of physical activity:				
Moderate–vigorous physical activity	Continuous	0.97	0.96–0.99	**0.03**
	Low	*reference*		
	Middle	0.80	0.61–0.95	**0.01**
	High	0.74	0.60–0.89	**0.02**
Physical workload	Continuous	1.02	1.01–1.05	**0.05**
	Low	*reference*		
	Middle	1.08	1.01–1.13	**0.05**
	High	1.11	1.03–1.24	**0.02**
By domain of physical activity:				
Leisure physical activity	Continuous	0.88	0.64–1.04	0.09
	Low	*reference*		
	Middle	0.65	0.50–1.01	0.06
	High	0.69	0.55–1.08	0.07
Transport physical activity	Continuous	0.97	0.90–1.09	0.09
	Low	*reference*		
	Middle	0.92	0.87–1.05	0.09
	High	0.83	0.79–0.89	0.08
Household physical activity	Continuous	1.04	0.98–1.13	0.07
	Low	*reference*		
	Middle	1.08	0.93–1.35	0.11
	High	1.10	0.94–1.27	0.09
Work physical activity	Continuous	1.01	0.91–1.55	0.08
	Low	*reference*		
	Middle	1.03	0.90–1.81	0.08
	High	1.06	0.92–1.63	0.06

*Notes*: Co‐variants adjusted for pain intensity, stress, disability, the presence of LBP at baseline, sleep quality, age and BMI.

Estimates in bold at significant at *p* < 0.05. Each type of physical activity was analysed as a separate model.

Abbreviations: 95% CI, 95% confidence interval; IRR, incidence risk ratios.

### 3.3 Physical activity and activity limitation associated with LBP

Results from the negative binomial models showed that increased sedentary time was significantly associated with an increased number of activity limitation counts for LBP (IRR 1.04, 95% confidence interval 1.01–1.09) (Table 3). These findings remained significant and the association increased in magnitude for the middle (IRR 1.07, 95% confidence interval 1.03–1.23) and high tertiles (IRR 1.15, 95% confidence interval 1.07–1.31) compared to the low reference tertile. For physical activity intensity, the negative binomial model showed no significant associations between moderate–vigorous physical activity or physical workload and the number of activity limitation counts (Table 3). For domain‐based physical activity measures, results from the negative binomial models showed significant associations only for leisure activity. Findings showed that increased engagement in leisure activity was associated with a lower number of activity limitation counts (IRR 0.94, 95% CI 0.81–0.99) (Table 3). These results were further established when participants in the middle (IRR 0.90, 95% CI 0.80–0.97), and in the high tertiles (IRR 0.89, 95% CI 0.81–0.99) were compared to those in the low reference tertile (Table 3). Additional analyses for the domains of transport, household and work activity did not demonstrate statistically significant associations across continuous measurements or tertiles.

**TABLE 3:** The relationship between intensity and domain of physical activity and activity limitation episodes for LBP (*n* = 140)

Explanatory variable	Volume	IRR	95% CI	*p*
Sedentary behaviour	Continuous	1.04	1.01–1.09	**0.04**
	Low	*reference*		
	Middle	1.07	1.03–1.23	**0.02**
	High	1.15	1.07–1.31	**0.01**
By intensity of physical activity:				
Moderate–vigorous physical activity	Continuous	0.91	0.88–1.21	0.08
	Low	*reference*		
	Middle	0.67	0.55–1.01	0.06
	High	0.62	0.47–1.09	0.11
Physical workload	Continuous	1.11	0.92–1.35	0.11
	Low	*reference*		
	Middle	1.04	0.90–1.27	0.10
	High	1.20	0.93–1.44	0.07
By domain of physical activity:				
Leisure physical activity	Continuous	0.94	0.81–0.99	**0.05**
	Low	*reference*		
	Middle	0.90	0.80–0.97	**0.04**
	High	0.89	0.81–0.99	**0.04**
Transport physical activity	Continuous	0.93	0.88–1.03	0.06
	Low	*reference*		
	Middle	0.99	0.91–1.33	0.11
	High	0.90	0.85–1.07	0.08
Household physical activity	Continuous	1.01	0.95–1.22	0.09
	Low	*reference*		
	Middle	1.01	0.93–1.55	0.15
	High	1.03	0.90–1.48	0.11
Work physical activity	Continuous	1.01	0.93–1.33	0.10
	Low	*reference*		
	Middle	1.18	0.88–1.77	0.13
	High	1.27	0.95–1.43	0.07

*Notes*: Co‐variants adjusted for pain intensity, stress, disability, the presence of LBP at baseline, sleep quality, age, and BMI.

Estimates in bold at significant at p < 0.05. Each type of physical activity was analysed as a separate model.

Abbreviations: 95% CI, 95% confidence interval; IRR, incidence risk ratios.

## 4 DISCUSSION

### 4.1 Summary of results

The aim of this study was to assess the relationship between different types (e.g. leisure, transport, household, work related), and intensities (e.g. moderate, vigorous) of physical activity assessed via the device and self‐reported questionnaires, and the frequency of analgesic use and activity limitation in people with LBP. Our results showed that different types and frequencies of physical activity were associated with different levels of analgesic use and activity limitation related to LBP. For analgesic use, undertaking work activities that involve higher physical workload tasks was associated with more frequent use of analgesics for LBP. Conversely, engaging in increased moderate–vigorous physical activity was associated with less frequent analgesic use counts for LBP. Additionally, we found that higher sedentary time was associated with a greater frequency of activity limitation counts for LBP and engaging in higher amounts of leisure time was associated with less frequency of activity limitation counts for LBP.

### 4.2 Findings compared to previous research

Sedentary time has been previously associated with a moderate increase in the risk of developing chronic LBP (OR 1.31, 95% CI 1.08–1.58) (Heneweer et al., 2009). Our results support and extend this finding, as sedentary time is shown to be significantly associated with an increased frequency of reports of activity limitation in people with LBP. This relationship was dose dependent and the occurrence of activity limitation counts increased as the sedentary time increased. Despite differences in the study populations, a study of active lifestyles in older people (Simmonds et al., 2014) found similar benefits in engagement in moderate–vigorous physical activity and analgesic use and health service utilization in seniors (Simmonds et al., 2014). Findings from this observational study indicated that engaging in higher moderate–vigorous physical activity was predictive of less frequent analgesic use (Simmonds et al., 2014), with people categorized as having high moderate–vigorous physical activity reporting 50% less prescriptions annually than those in the low and moderate–vigorous physical activity groups (Simmonds et al., 2014). Although the two studies recorded analgesic use differently, together these studies strengthen the potential role of moderate–vigorous physical activity in supporting older people or those with LBP to reduce the frequency of analgesic use.

Previous research has shown conflicting evidence on the association between occupational physical activities and LBP (Kwon et al., 2011b). We found that increased time and frequency of physical workload tasks were associated with an increased frequency of reports of analgesic use for LBP, a finding that is in agreement with the results from recent studies (Coenen et al., 2014; Heuch et al., 2017; Sterud & Tynes, 2013). While analgesic use was not an outcome measured in these studies, they showed that engaging in more strenuous physical work (involving bending, twisting, lifting, pushing or pulling) increased the relative risk of people developing chronic LBP by 30% compared to those with sedentary work (Coenen et al., 2014; Heuch et al., 2017; Sterud & Tynes, 2013).

Existing literature has proposed that engaging in leisure time physical activity can be protective against chronic LBP (Shiri & Falah‐Hassani, 2017; Solovev et al., 2020), however, this association may follow a U‐shaped curve (Solovev et al., 2020). Our findings differ from this, as we found that in people with LBP, those participating in higher amounts of leisure‐time physical activity presented with less frequent counts of activity limitation. In fact, our findings indicate a potential linear relationship between leisure‐time physical activity and frequency of activity limitation in people with LBP, as associations increased both when people in the middle and high leisure time groups were compared with those in the low leisure time reference group.

### 4.3 Strengths and limitations

Our study had several strengths. The use of a cohort design allowed for associations between exposures and outcomes to be quantified (Rezigalla, 2020; Sedgwick, 2013) and to identify specific factors that were potential predictors of the outcome (Rezigalla, 2020; Sedgwick, 2013). Potential factors affecting the association between physical activity and LBP (such as depression, anxiety, stress, sleep quality, pain intensity and duration, disability, BMI, gender and age) were recorded at baseline and accounted for in the negative binomial regression models (Rezigalla, 2020; Sedgwick, 2013). Device‐based data on physical activity intensity were collected via accelerometry, which has shown to be one of the most valid, reliable, accurate and sensitive instruments in assessing physical activity (Colbert et al., 2011; Ellis et al., 2014; Warren et al., 2010). Device‐based data were supplemented by validated and commonly used self‐reported tools, such as the IPAQ‐long form, to assess physical activity participation across a variety of domains.

This study presents some limitations that should be taken into consideration. The sample sizes of 86 individuals taking analgesics and 140 individuals with activity limitations are small compared to most prospective cohort studies. Finding significant results with small sample sizes increases the potential for the effect sizes to be inflated (Button et al., 2013). Therefore, our results should be interpreted with some caution and replicated with larger samples. Additionally, despite the accurate information accelerometers can provide about levels and patterns of physical activity, they do not record postures, and, consequently, sedentary time measures may include standing time (O'Brien et al., 2020; Troiano et al., 2008). It is also known that subjective measures of physical activity can result in under‐ and overestimation of the amount of physical activity reported, as answers are subject to recall bias (Fogelholm et al., 2006; Kremer et al., 1981; Rzewnicki et al., 2003; van Weering et al., 2011). Additionally, our study sample included individuals with and without a history of LBP at baseline, therefore, there is limited ability to infer causation from our study findings. However, this potential confounding effect is lessened due to the adjustment for the presence of LBP at baseline in our analysis.

### 4.4 Clinical implications

Our results highlight the importance of supporting people with LBP to engage in moderate–vigorous and leisure physical activity, minimize sedentary time and time spent on activities involving high physical workload tasks to reduce the need for analgesic use and the risk of activity limitation. An example of a leisure activity that can be moderate–vigorous in intensity is walking. Walking is known to be a safe form of moderate–vigorous physical activity for individuals with LBP (Hurley et al., 2009) and is associated with a low injury rate and does not involve twisting or vigorous forward flexion (Hurley et al., 2009). Existing evidence has also highlighted that individuals with LBP can minimize sedentary time through self‐monitoring tools, restructuring the physical environment and social accountability (Lansing et al., 2021). Additionally, raising awareness of the physical strains that frequently occur during occupational work, changing work practices and redesigning the work environment have shown to be effective at reducing physical workload tasks for individuals with LBP (Kozak et al., 2017). Therefore, clinicians should promote interventions and lifestyle changes that allow individuals with LBP to engage in the aforementioned physical activity domains and intensities that reduce the need for analgesic use and the risk of activity limitation. By doing so, the costly and disabling burden of analgesic use and activity limitation as a result of LBP imposed on individuals and societies can be lessened.

### 4.5 Directions for future research

Future research in the form of randomized control trials should investigate the degree of certainty for the potential linear relationship found between leisure‐time physical activity and sedentary time on the frequency of activity limitation in people with LBP. Additionally, the potential dose‐dependent relationship between physical workload and moderate‐vigorous physical activity on analgesic use by people with LBP should also be examined with appropriately powered groups and objective measures. We also acknowledge the potential for analysis to be sub‐grouped across different types of analgesics and the severity of activity limitation.

## 5 CONCLUSION

Increased time spent on sedentary activities and high physical workload tasks are associated with more frequent reports of analgesic usage and activity limitation in people with LBP over a 1‐year period. Conversely, increasing time spent on moderate–vigorous and leisure physical activity might be protective for future analgesic usage and reports of activity limitation. These results support the implementation of lifestyle‐based interventions as support systems to empower people with LBP to minimize activity limitations and reduce their reliance on analgesics.

## AUTHORS’ CONTRIBUTION

I hereby declare that this submission is my own work and that it contains no material previously published or written by another person except where acknowledged in the text. Thomas G. Patterson: conceptualization, data curation, formal analysis, investigation, methodology, project administration, resources, software, validation, visualization, writing – original draft and writing – review and editing. Paula R. Beckenkamp: conceptualization, data curation, formal analysis, investigation, methodology, project administration, resources, software, supervision, validation, visualization and writing– review and editing. Manuela Ferreira: conceptualization, data curation, formal analysis, investigation, methodology, project administration, resources, software, supervision, validation, visualization and writing– review and editing. Adrian Bauman:conceptualization, data curation, formal analysis, investigation, methodology, project administration, resources, software, supervision, validation, visualization and writing – review and editing. Ana Carvalho de Silva: conceptualization, data curation, formal analysis, investigation, methodology, project administration, resources, software, supervision, validation, visualization and writing – review and editing. Lucas Calais Ferreira: conceptualization, data curation, formal analysis, investigation, methodology, project administration, resources, software, supervision, validation, visualization and writing – review and editing. Paulo H. Ferreira: conceptualization, data curation, formal analysis, investigation, methodology, project administration, resources, software, supervision, validation, visualization and writing – review and editing.

## CONFLICT OF INTERESTS

All authors have completed the ICMJE Conflicts of Interest form, declaring no conflicts of interest with other people or organizations, that impact this submitted work.
